# Rapid and improved oral absorption of N-butylphthalide by sodium cholate-appended liposomes for efficient ischemic stroke therapy

**DOI:** 10.1080/10717544.2021.2000678

**Published:** 2021-11-12

**Authors:** Ailing Zhang, Jianbo Li, Shuaishuai Wang, Yaru Xu, Qinglian Li, Zhe Wu, Chenxu Wang, Haiyang Meng, Jinjie Zhang

**Affiliations:** a Department of Pharmacy, The First Affiliated Hospital of Zhengzhou University, Zhengzhou, China; b Henan Key Laboratory for Pharmacology of Liver Diseases, Institute of Medical and Pharmaceutical Sciences, Zhengzhou University, Zhengzhou, China; c Henan Key Laboratory of Targeting Therapy and Diagnosis for Critical Diseases, School of Pharmaceutical Sciences, Zhengzhou University, Zhengzhou, China

**Keywords:** Ischemic stroke, rapid absorption, brain accumulation, drug release, N-butylphthalide

## Abstract

As a multi-target drug to treat ischemic stroke, N-butylphthalide (NBP) is extremely water-insoluble and exhibits limited oral bioavailability, impeding its wide oral application. Effective treatment of ischemic stroke by NBP requires timely and efficient drug exposure, necessitating the development of new oral formulations. Herein, liposomes containing biosurfactant sodium cholate (CA-liposomes) were systemically investigated as an oral NBP delivery platform because of its high biocompatibility and great potential for clinical applications. The optimized liposomes have a uniform hydrodynamic size of 104.30 ± 1.60 nm and excellent encapsulation efficiency (93.91 ± 1.10%). Intriguingly, NBP-loaded CA-liposomes produced rapid drug release and the cumulative release was up to 88.09 ± 4.04% during 12 h while that for NBP group was only 6.79 ± 0.99%. Caco-2 cell monolayer assay demonstrated the superior cell uptake and transport efficiency of NBP-loaded CA-liposomes than free NBP, which was mediated by passive diffusion via transcellular and paracellular routes. After oral administration to rats, NBP-loaded CA-liposomes exhibited rapid and almost complete drug absorption, with a t_max_ of 0.70 ± 0.14 h and an absolute bioavailability of 92.65% while NBP suspension demonstrated relatively low bioavailability (21.7%). Meanwhile, NBP-loaded CA-liposomes produced 18.30-fold drug concentration in the brain at 5 min compared with NBP suspension, and the brain bioavailability increased by 2.48-fold. As expected, NBP-loaded CA-liposomes demonstrated significant therapeutic efficacy in a middle cerebral artery occlusion rat model. Our study provides new insights for engineering oral formulations of NBP with fast and sufficient drug exposure against ischemic stroke in the clinic.

## 1. Introduction

Ischemic stroke, a lethal cerebrovascular disease, was considered as one of the leading causes of death worldwide (Silva & Schwamm, 2021). However, there is no complete cure for ischemic stroke in the clinic (Zhou et al., 2018). The primary therapeutic options involving thrombolysis and neuroprotection, only partially improve symptoms and increase survival (Moretti et al., 2015). Specifically, thrombolytic therapy can restore the blood flow in the brain but its application is limited by the risk of ischemia-induced neurovascular damage as well as the narrow therapeutic window (Zhou et al., 2018). Another therapeutic strategy is focused on developing neuroprotective agents to prevent damage of nerve cells caused by hypoxia and ischemia. Notwithstanding, multiple clinical trials on presumed neuroprotectants have failed (Zhou et al., 2018). Owing to complicated pathophysiology of ischemic stroke and lack of blood-brain barrier (BBB) penetration, these drugs are difficult to exert their therapeutic effect via blocking a single pathological process (Neuwelt et al., 2008).

N-butylphthalide (NBP), a natural oily compound extracted from celery seeds, has been approved by the National Medical Products Administration of China for the treatment of ischemic stroke (Wang et al., 2014). Numerous studies have reported that NBP exhibited multi-target neuroprotective effects on ischemia such as decreasing brain edema, improving microcirculation in the ischemic region, inhibiting oxidative stress, suppressing apoptosis as well as protecting the BBB (Chen et al., 2020; Li et al., 2021; Liu et al., 2021; Wang et al., 2021). NBP sodium chloride injection using HP-β-CD as a drug solubilizer is already available in the market and demonstrates efficient stroke treatment as highly lipophilic NBP realized good penetration through BBB (Chen et al., 2017). Nevertheless, orally delivered NBP would be more attractive for patients, owing to higher medical convenience and adherence (Ahadian et al., 2020). However, the development of oral drug delivery systems for NBP was rarely reported (Wang et al., 2015). As the only marketed product of oral NBP formulation in China, NBP soft capsules possess significant clinical efficacy in the treatment of ischemic stroke but still suffer from low oral bioavailability (Ye, 2004; Zhang et al., 2017; Wang et al., 2020). NBP is poorly water soluble and exhibited extremely slow dissolution in the gastrointestinal tract, as revealed in the present study. The unfavorable physicochemical properties of NBP inevitably lead to inefficient oral absorption and poor brain accumulation, which result in undesired efficacy for the treatment of ischemic stroke *in vivo*. Therefore, oral formulations of NBP providing timely and sufficient brain accumulation are urgently needed for effective treatment of ischemic stroke.

Despite the rapid development of drug delivery platforms, liposomes remain the most promising drug delivery system with good biocompatibility and absorption-enhancing capability (Filipczak et al., 2020). To date, several liposomal drugs have reached the market for treating various diseases (Jensen & Hodgson, 2020). Recently, addition of bile salts upon liposome formation was found to make the generated liposomes more flexible, thereby promoting the vehicles across cell absorption barrier (Niu et al., 2014). Moreover, compared with conventional liposomes, bile salts-appended liposomes demonstrated significantly higher drug loading capacity and oral bioavailability (Chen et al., 2009; Thakur et al., 2012; Arafat et al., 2017). Bile salts-appended liposomes have also been successfully utilized to increase the dissolution rate of drug with poor solubility in the gastrointestinal (GI) tract (Dai et al., 2013; Faustino et al., 2016). More importantly, bile salts are beneficial to the absorption of lipid-soluble compounds *in vivo* (Aburahma, 2016; Macierzanka et al., 2019). Taken together, bile salts-appended liposomes show great promise in overcoming the above issues related to oral delivery of NBP in treating ischemic stroke.

To date, encapsulation of NBP into bile salts-appended liposomes has not been reported. Sodium cholate (CA), a naturally occurring bile acid in humans, shows strong solubilization capacity and significant absorption enhancement of water insoluble drugs (Maswal and Dar, 2013; Cona et al., 2015). Herein, we developed a new oral NBP formulation using liposomes containing CA (NBP-loaded CA-liposomes), and explored its potential effects on the drug delivery performance of NBP. To this end, *in vitro* and *in vivo* studies were conducted to investigate the physicochemical properties, absorption mechanisms, pharmacokinetics, biodistribution as well as therapeutic outcomes of NBP-loaded CA- liposomes.

## 2. Materials and methods

### 2.1. Materials

NBP and egg yolk phosphatidylcholine (PC-98T) were purchased from Sigma-Aldrich Co., Ltd. (Shanghai, China) and Aiweituo Pharmaceutical Technology Co., Ltd. (Shanghai, China), respectively. Sodium cholate (CA) was bought from Aladdin Biochemical Technology Co., Ltd. (Shanghai, China). All solvents were provided by Hengxing Chemical Reagent Manufacturing Co., Ltd. (Tianjin, China), unless otherwise stated. Malondialdehyde, superoxide dismutase and glutathione peroxidase assay kits were provided by Jiancheng Technology Co., Ltd. (Nanjing, China).

### 2.2. Preparation and optimization of NBP-loaded sodium cholic-appended liposomes (NBP-loaded CA-liposomes)

Thin film-hydration method was applied for the preparation of liposomes as previously reported (Niu et al., 2014). Specifically, NBP, PC-98T and CA at predetermined weight ratios were completely dissolved in anhydrous ethanol in a round-bottom flask. The solvent was then removed by reduced pressure to form a uniform lipid film on the flask wall followed by complete hydration with 5 mL of double-distilled water. The obtained dispersion was homogenized using an ultrasonic disintegrator (JY99-II DN, Xinzhi Biotechnology Inc.) for 5 minutes in pulse mode (5 s on; 10 s off) in an ice bath. Afterwards, the sample was centrifuged (3,000 rpm, 3 min) and the upper liposome dispersion was collected as NBP-loaded CA-liposomes. Finally, NBP-loaded CA-liposomes were optimized via evaluating the effects of the weight ratio of PC-98T/CA on the physicochemical properties (See section 2.4) of generated liposomes.

### 2.3. HPLC analysis

The concentrations of NBP present in the harvested samples were analyzed by an Agilent 1200 HPLC system. Specifically, chromatographic separation was achieved by an Agilent analytical column (Zorbax Eclipase XDB-C18, 4.6 × 150 mm, 5 µm), while the mobile phase consisted of 0.2% aqueous phosphoric acid and acetonitrile (50/50, v/v) was used at a flow rate of 1.0 mL/min. The NBP concentration was monitored at 230 nm and calculated from linear standard curves with an injection volume of 20 µL. All measurements were done in triplicate.

### 2.4. Characterizations of NBP-loaded CA-liposomes

The physicochemical properties of liposomes prepared at different weight ratio of PC-98T/CA were investigated. The average diameter and zeta potential of the samples were measured by a Zetasizer Nano ZS90 instrument (Malvern, UK) after suitable dilution with double distilled water (1:50, v/v). The concentration of NBP in NBP-loaded CA-liposomes was assessed by HPLC to determine drug encapsulation efficiency (EE) and drug loading capacity (DL). The samples were processed using an ultrafiltration method as previously reported (Li et al., 2018). Briefly, to analyze the content of free NBP (W_f_), 0.5 mL liposome was ultrafiltrated and the filtrate was collected and subjected to HPLC. To analyze the total added amount of NBP (W_t_), 0.1 mL liposomes were diluted with 2 ml of methanol and then sonicated and centrifugated (10,000 rpm, 20 minutes). Afterwards, the supernatant was injected into the HPLC system. The amount of entrapped drug was obtained by subtracting the amount of free drug from the total drug. The equations used to calculate EE and DL were listed as follows:

(1) $$\text{EE}\%=\frac{\left(W_{t}-W_{f}\right)}{W_{t}}\times 100\%$$

(2) $$\text{DL}\%=\frac{W_{t}-W_{f}}{\text{weight}\;\text{of}\;\text{the}\;\text{excipients}\;\text{and}\;\text{drug}}\times 100\%$$

Where W_f_ is the mass of free NBP, W_t_ is the total amount of NBP in the liposomes.

The size and morphology of negative-stained liposomes with optimum attributes were further visualized under a transmission electron microscope (TEM, JEM-1200EX). Meanwhile, colloidal stability upon dilution was evaluated by monitoring the changes of vesicle size as the degree of dilution increased from 10 times to 200 times. Finally, the storage stability assessment were performed at 4 °C for 2, 4, 6, 7, 15, 30 d, followed by measurements of vesicle size, zeta potential and entrapment efficiency, respectively.

### 2.5. *In vitro* drug release from NBP-loaded CA-liposomes

The release curves of the formulations were obtained using a dialysis method (Zhang et al., 2011). To begin with, 0.5 mL of freshly prepared NBP suspension in water and NBP-loaded CA-liposomes were sealed into dialysis bags (molecular weight cutoff 5,000), accordingly. Thereafter, the dialysis bags were incubated at 37 °C in 50 mL of simulated gastric fluid (SGF, pH 1.2) and shaken at 100 rpm. 1 mL of the release medium was taken out at various times (10 min, 20 min, 30 min, 1 h and 2 h) for HPLC analysis and replenished with fresh media of equal volume. Thereafter, the dialysis bags were transferred into phosphate buffer solution (pH 6.8) of equal volume, and 1 mL of release medium was collected after another 2, 4, 6 and 10 h. The following procedures were performed as above.

### 2.6. Investigation of absorption mechanism of NBP-loaded CA-liposomes in caco-2 cells

To elucidate the absorption mechanism of NBP-loaded CA-liposomes, cell viability, cellular uptake and permeability of NBP-loaded CA-liposomes across the Caco-2 cell monolayers were examined. The cells were cultured as described in previously reported protocols (Li et al., 2019). Cytotoxicity of NBP, blank liposomes (containing CA but without NBP) and NBP-loaded CA-liposomes were evaluated by MTT assay after incubation with Caco-2 cells for 4 h. Finally, absorbance at λ = 490 nm were measured by a microplate reader. The results were normalized and expressed as the percentage of absorbance of the untreated cells.

For the uptake tests, Caco-2 cells were plated in 6-well plates followed by incubation with NBP solution and NBP-loaded CA-liposomes at a NBP concentration of 800 µmoL/L (37 °C, 2 h). Thereafter, total protein content of the cells per well was measured by a BCA kit upon freeze and thaw lysis for three cycles while the amount of NBP taken up by the cells was determined by HPLC and normalized to the total protein amount. To explore whether the uptake of free NBP and NBP-loaded CA-liposomes was energy-dependent, we performed further cell uptake assay at 4 °C or in the presence of NaN_3_ followed by treatment procedures as above.

For the transport permeability tests, Caco-2 cell monolayer models were well established following a previously reported method (Li et al., 2019). Millicell electrical resistance equipment (Millipore, USA) was used to monitor the transepithelial electrical resistance (TEER) values of cell monolayers. When a TEER value over 800 cm Ω was detected, the monolayer was subjected to the following studies. To begin with, 1.5 mL of fresh Hanks balanced salt solution were added to the basolateral side while 500 μL of NBP solution or NBP-loaded CA-liposomes (400 µmoL/L) were added to the apical side of the well-formed monolayer at 37 °C, respectively. The permeability ability of NBP was assessed by HPLC analysis of samples taken from BP side at various times. To explore whether the permeation of free NBP and NBP-loaded CA-liposomes was energy-dependent, we performed further transport study at 4 °C or in the presence of NaN_3_. To study the influence of CA in liposomes on the tight junctions of Caco-2 cell monolayers, conventional liposomes, blank CA-liposomes, and NBP-loaded CA-liposomes were incubated with Caco-2 cell monolayer and TEER was monitored at different time points. P_app_ values of NBP were calculated according to the equation:

$$P_{\text{app}}=\left(\text{dQ}⁄\text{dt}\right)⁄{\text{A C}}_{0}$$

(3)




Where the dQ/dt (μg/s) is the permeation rate of drug, A is the membrane area (cm^2^) and C_0_ (μg/mL) is the drug concentration at time 0 (31).

### 2.7. Pharmacokinetics studies

Male Sprague-Dawley rats (250 ± 20 g) were obtained from the laboratory animal center of Zhengzhou University (Zhengzhou, China). The animal experiments were conducted in accordance with the Regional Ethics Committee and protocols from the Institutional Animal Care and Use Committee of Zhengzhou University. Rats were fasted overnight with free access of water before the pharmacokinetic studies. To begin with, rats were randomly assigned into three groups. One group was intravenously received NBP solution (dissolved in 20% Solutol HS 15) at a dose of 30 mg/kg while the other two groups were orally given NBP suspension and NBP-loaded CA-liposomes at an equivalent dose, respectively. 0.5 mL of blood was taken from rats by retro-orbital sinus at 0.083, 0.25, 0.5, 1, 2, 3, 4, 6, 8, and 10 h after administration, respectively. The concentration of NBP in plasma was determined by HPLC after suitable processing. Briefly, 300 µL methanol was mixed with 100 µL of plasma and the resultant suspension was centrifuged (12,000 rpm, 10 min). The supernatant obtained was analyzed by a validated HPLC method (Supplementary Table 2). The pharmacokinetic parameters were obtained using DAS 3.0 software (Mathematical Pharmacology Professional Committee of China) and were presented as mean ± SD. The absolute bioavailability (F_a_) and the relative bioavailability (F_r_) were calculated by the following equations, respectively:

(4) $$\mathrm{Fa}\;(\%)=\frac{{\text{AUC}}_{0-t,\;\text{oral}}{\text{Dose}}_{i.v}}{{\text{AUC}}_{0-t,\;i.v}{\text{Dose}}_{\text{oral}}}$$

(5) $$\mathrm{Fr}\;(\%)=\frac{{\text{AUC}}_{0-t,\;\text{formulation}}}{A{\text{UC}}_{0-t,\;\text{NBP}\;\text{suspension}}}$$

Where AUC_0−t_ represents the area under the pharmacokinetic curve throughout the study.

### 2.8. Biodistribution studies

Before the start of the experiment, rats were fasted while allowed to drink freely overnight. NBP solution and NBP-loaded CA-liposomes were orally administered to rats giving a dosage of 30 mg/kg per rat. Rats were sacrificed 0.083, 0.25, 0.5, 1 and 2 h post-administration, and subsequently, the vital organs involving heart, liver, spleen, kidney, lung as well as brain were harvested and processed by following procedures. In brief, the harvest tissues were weighed and homogenized in cold saline. The obtained tissue lysates (100 μL) was methanol precipitated and centrifuged (4 °C, 15 min) followed by injection of the supernatant (50 μL) into HPLC. The NBP concentration in each tissue sample was calculated from the respective standard curves. To investigate the brain-accumulation properties of NBP-loaded CA-liposomes, the key targeting indexes of major tissues involving the relative uptake efficiency (Re) and concentration efficiency (Ce) were calculated as follows (Li et al., 2018).$Re=\;\frac{\text{AU}C_{0-t,\;\text{tissue},\;\text{formulation}}}{\text{AU}C_{0-t,\;\text{tissue},\;\text{NBP}\;\text{suspension}}}$(Wang et al., 2014)$Ce=\frac{C_{\text{max},\;\text{tissue},\;\text{formulation}}}{C_{\text{max},\;\text{tissue},\;\text{NBP}\;\text{suspension}}}$ (Wang et al., 2021)

### 2.9. Therapeutic studies on the cerebral ischemia/reperfusion (I/R) model in rats

Rats (280–320 g) were divided into five groups randomly (*n* = 10 per group): (Silva and Schwamm, 2021) sham (Normal saline), (Zhou et al., 2018) I/R, (Moretti et al., 2015) I/R + NBP (30 mg/kg), (Zhou et al., 2018) I/R + NBP-loaded CA-liposomes (30 mg/kg), (Neuwelt et al., 2008) I/R + blank liposomes. As reported previously, a rat middle cerebral artery occlusion (MCAO) method was used to establish I/R model as previously reported (Shi et al., 2020). Briefly, rats were anesthetized with 1% pentobarbital (45 mg/kg) and then fixed in the supine position. After lateral neck incision, the internal carotid artery, right common carotid artery (MCA) and external carotid artery were exposed. A surgical nylon suture was gently advanced to occlude MCA until slight resistance was felt (18–20 mm from MCA bifurcation). 1.5 hours post MCAO, reperfusion was inducted by taking out the suture and the rats were immediately orally given the predetermined formulations. The sham operated group received the same surgery in the presence of the MCAO and drug treatment. The core body temperature was kept constant (37 ± 0.5 °C) for 24 h post reperfusion. Neurological deficit of rats was then assessed by the blinded Longa’s method. To observe the infarcts of rats, some rats (*n* = 6) from each group were decapitated to harvest 2-mm-thick coronal brain slices and these slices were then incubated with 2% TTC solution (37 °C, 15 min). The cerebral infarct area (White) was analyzed by Image-J and represented as a percentage of the total brain area. Histological alterations of brain samples from the remaining rats were analyzed via hematoxylin and esosin (H&E) staining and then observed under a microscope. The levels of malondialdehyde (MDA), superoxide dismutase (SOD) and glutathione peroxidase (GSH-Px) in brain lysates were determined 24 h after reperfusion according to the protocols of the specific agent kit (Servicebio, Wuhan, China).

### 2.10. Statistical analysis

All results were reported as mean ± standard deviation (SD). Statistical analysis between was made by two tail Student’s *t*-test and one-way ANOVA, and the differences were considered to be statistically significant at *p* < .05.

## 3. Results and discussion

Despite that numerous liposome formulations have been successfully utilized to enhance the pharmacokinetic as well as pharmacodynamic outcomes of drugs (Belfiore et al., 2018; Joshi et al., 2019), effective liposome platform for NBP remains lacking. Our group attempted to load NBP within conventional liposomes, but failed to achieve efficient drug load. This was partially explained by the fact that NBP was an oily compound with extremely poor water solubility (Li et al., 2018). Previous studies found that the bile salts-appended liposomes significantly increased drug loading efficiency and improved oral absorption relative to conventional liposomes (Yang et al., 2015; Aburahma, 2016). This led us to explore the beneficial effect of NBP-loaded CA-liposome for ischemic stroke therapy.

### 3.1. Preparation and characterizations of NBP-loaded CA-liposomes

**Figure 1.**
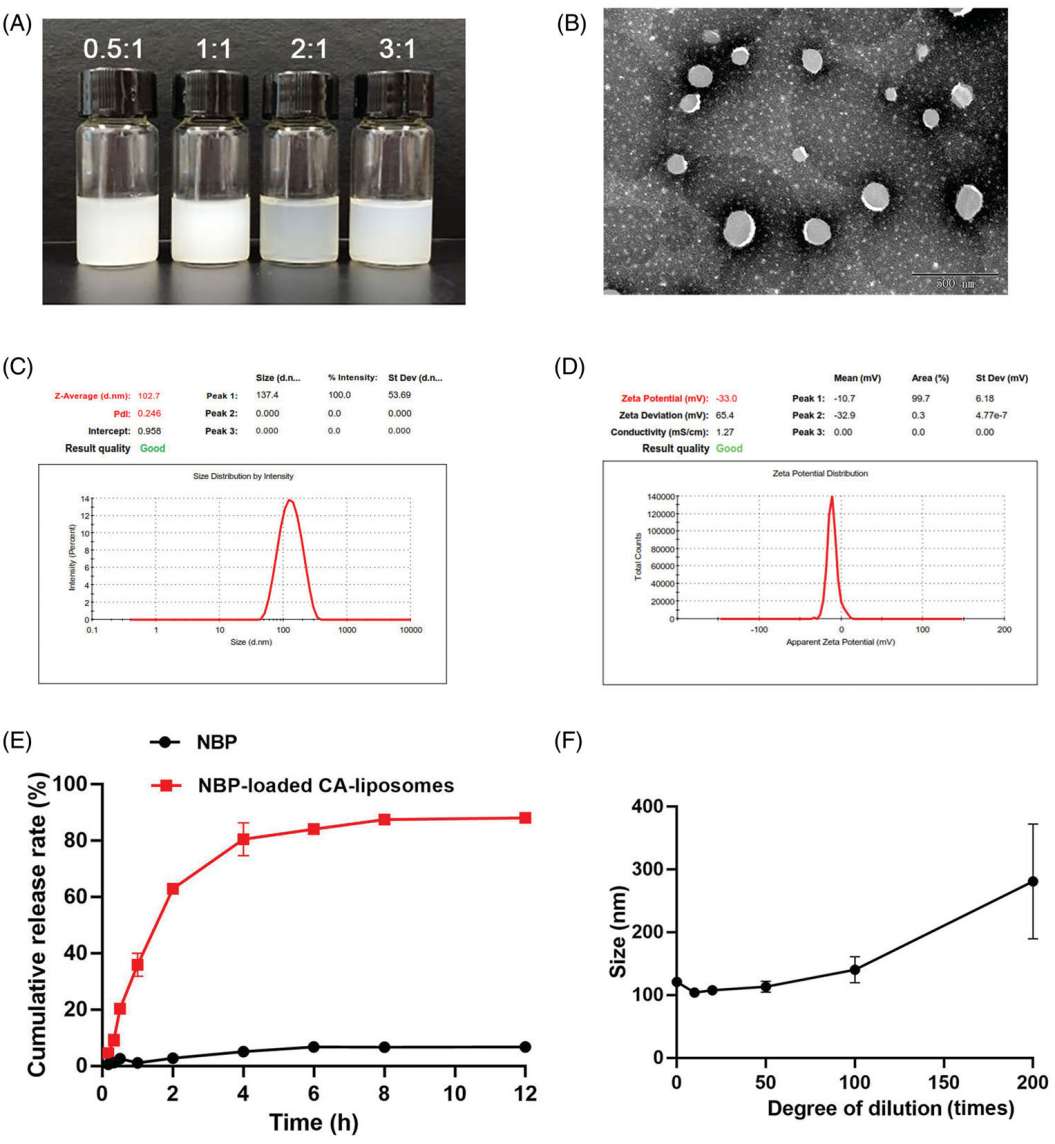
Characterizations of NBP-loaded CA-liposomes. (A) Representative photos of NBP-loaded CA-liposomes prepared at PC-98T/CA ratios = 0.5:1, 1:1, 2:1 and 3:1, respectively. (B) Representative TEM image, (C) Particle size distribution and (D) Zeta potential of the optimized NBP-loaded CA-liposomes prepared at the PC-98T/CA ratio of 3:1. (E) *In vitro* NBP release profiles from free NBP solution and NBP-loaded CA-liposomes. (F) Size changes of NBP-loaded CA-liposomes that were diluted 10 times to 200 times with de-ionized water. Data are mean ± SD (*n* = 3).

In this work, NBP-loaded CA-liposome was prepared using a thin film dispersion method (Lei et al., 2019). We first optimized the NBP-loaded CA-liposome via evaluating the effects of the weight ratio of PC-98T/CA on the vesicle size, zeta potential, DL and EE. Figure 1(A) shows the photos of NBP-loaded CA-liposomes that were prepared at the PC-98T/CA ratios of 0.5:1, 1:1, 2:1 and 3:1, respectively. Their physico-chemical properties were presented in Table 1. DL and EE increased markedly as the PC-98T/CA ratio increased. Notably, liposomes consisted of PC-98T and CA at a weight ratio of 3:1, providing an average hydrodynamic diameter at around 104.30 ± 1.60 nm with a relatively uniform size distribution while its surface charge was −32.30 ± 4.38 mV (Table 1). Meanwhile, the DL and EE of the liposomes were 8.48 ± 0.10% and 93.91 ± 1.10%%, which were highest among all the tested groups. Therefore, it was selected as the optimum formulation and investigated in the following studies.

**Table 1.** Effect of PC-98T/CA ratio on the physicochemical properties of NBP-loaded CA-liposomes (mean ± SD, *n* = 3).

PC-98T/CA (weight ratio)	Size (nm)	PDI	Zeta potential (mV)	DL (%)	EE (%)
0.5:1	103.9 ± 46.88	0.428 ± 0.141	−1.33 ± 1.23	4.73 ± 0.06	50.97 ± 0.64
1:1	179.0 ± 85.99	0.361 ± 0.055	−0.64 ± 1.77	6.55 ± 0.06	70.19 ± 1.08
2:1	90.95 ± 3.86	0.630 ± 0.036	−33.3 ± 4.01	7.97 ± 0.20	85.99 ± 2.15
3:1	104.3 ± 1.60	0.259 ± 0.012	−32.3 ± 4.38	8.48 ± 0.10	93.91 ± 1.10

The morphology of the optimized liposomes was observed under TEM and shown in Figure 1(B). After negative staining, liposomes demonstrated a bright and uniform spherical structure. Figure 1(C,D) clearly shows the vesicle size and zeta potential distribution of the optimized liposomes, which are in accordance with the results shown in Table 1. The drug release curves of NBP suspension and NBP-loaded CA-liposomes were illustrated in Figure 1(E). Drug release from NBP suspension was very slow and its cumulative drug release amount was about 6% within 2 hours. In contrast, NBP-loaded CA-liposomes showed rapid drug release and the cumulative release was 62.98 ± 1.04% within 2 h, indicating remarkably improved drug dissolution rate. The 24 h cumulative drug release amount from NBP suspension and NBP-loaded CA-liposomes were 6.79 ± 0.99% and 88.09 ± 4.04%, respectively. Compared with NBP, NBP-loaded CA-liposomes exhibited dramatically faster drug release *in vitro*, which was beneficial to timely and efficient absorption of NBP for treating ischemic stroke. To tentatively predict the stability of liposomes once arrived at gastrointestinal tract, the change of NPs size upon dilution was monitored. No obvious size change was observed in the samples that were diluted 50-times with double distilled water (Figure 1(F)), indicating minimum effect on the NPs after dilution by gastrointestinal fluids. In addition, storage stability of NBP-loaded CA-liposomes at 4 °C was investigated by measurement of size, PDI, zeta potential and EE. As shown in Supplementary Table 1, NBP-loaded CA-liposomes was stable for at least one week, indicating good storage stability which was beneficial for application.

### 3.2. Cytotoxicity, intracellular uptake and uptake mechanism studies

**Figure 2.**
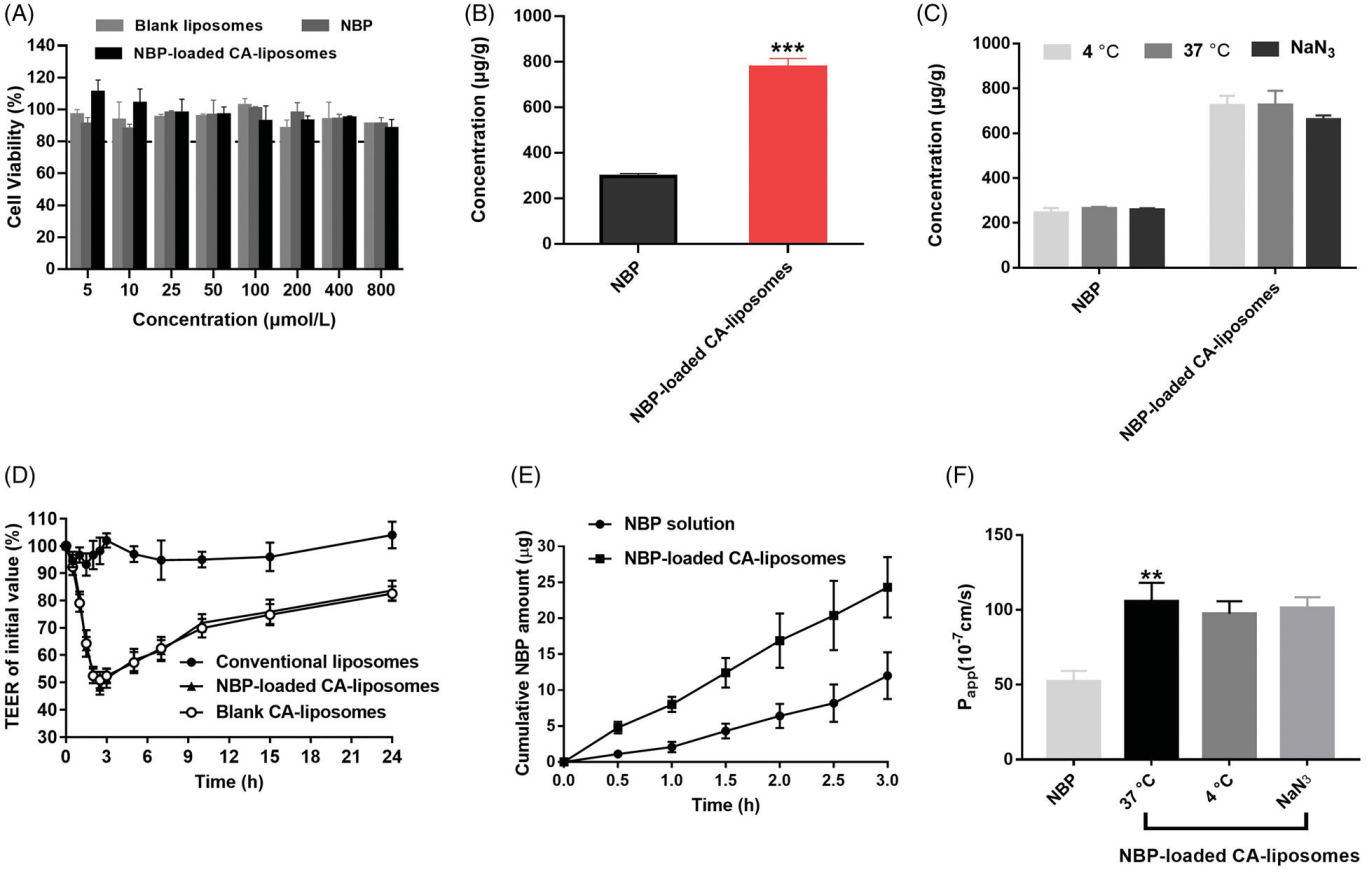
Cytotoxicity, cellular uptake and mechanistic studies. (A) The cytotoxicity of NBP and NBP-loaded CA-liposomes after incubation with Caco-2 cells for 4 h at different concentrations (5–800 µmoL/L), as determined by MTT assays. (B) Cellular uptake of NBP and NBP-loaded CA-liposomes at the drug concentration of 800 µmoL/L. (C) Cellular uptake assay at 4 °C or in the presence of NaN_3_, compared with that at 37 °C. (D) Effect of liposomes on TEER values of Caco-2 cell monolayers during 24 h incubation. Cumulative amounts (E) and P_app_ values of NBP (F) across Caco-2 cell monolayers after incubation with different formulations. Data are mean ± SD (*n* = 3). **p<.01, ***p< .001, compared with NBP solution.

As shown in Figure 2(A), no significant cytotoxicity on the Caco-2 cells was observed in all the tested groups at the concentration from 5 µmol/L to 800 µmol/L as demonstrated by MTT assay (*p*> .05). Subsequently, cell uptake studies demonstrated that NBP-loaded CA-liposomes significantly increased the cellular uptake of NBP by 1.5-fold compared with free NBP solution (Figure 2(B)). Hence, we sought to explore whether the uptake of NBP-loaded CA-liposomes by the Caco-2 cells occurred through energy-dependent pathways. Interestingly, the cellular uptake efficiency of NBP-loaded CA-liposomes did not vary significantly after incubation for 2 h at 4 °C or with NaN_3_ (*p*> .05, Figure 2(C)), indicating that the cell uptake process was energy-independent. Previous studies have proposed that cell internalization of liposomes was through energy-independent pathways such as clathrin or caveolin-mediated endocytosis (He et al., 2019; Filipczak et al., 2020). In the present study, we found that NBP-loaded CA-liposomes exhibited efficient cell uptake which was independent of energy. These findings pushed us to examine the possible transport mechanisms underlying the absorption behavior of NBP-loaded CA-liposomes using the Caco-2 monolayer cell model.

Given that liposomes containing bile salts were reported to improve drug absorption via reversibly opening tight junctions of intestinal epithelium (Niu et al., 2014), TEER value of the cell monolayers was monitored after incubation with blank CA-liposomes, conventional liposomes and NBP-loaded CA-liposomes at various time points. As shown in Figure 2(D), the fluctuation of TEER value was negligible in the conventional liposomes group during 24 h. By contrast, NBP-loaded CA-liposomes and blank CA-liposomes induced dramatically decreased TEER value at 2 h and then gradually increased the TEER value to approximately 80% of the initial value. These results implied that CA caused reversible opening of tight junctions, which may help NBP-loaded CA-liposomes enhance drug absorption *in vivo*. To evaluate the cell permeability of NBP-loaded CA-liposomes, the cumulative NBP amount across the Caco-2 cell monolayer during 3 h was determined and shown in Figure 2(E). Compared with NBP solution, NBP-loaded CA-liposomes exhibited significantly increased cumulative drug amount within 3 h (**p < .01), indicating their excellent cell penetration. Additionally, the P_app_ value of NBP-loaded CA-liposomes was significantly higher than that of NBP group (**p < .01, Figure 2(F)). Meanwhile, no significant difference was observed among the P_app_ values of NBP-loaded CA-liposomes at 4 °C, 37 °C and in the presence of NaN_3_ (*p*> .05)_,_ indicating that the transport process was energy-independent. Combined with the results of *in vitro* release studies showing that roughly 50% NBP released from the liposomes within 2 h (Figure 1(E)), we speculated that the enhanced cell absorption was mediated by passive membrane penetration of released NBP via paracellular and transcellular routes but not the internalization of intact liposomes.

### 3.3. Pharmacokinetic studies

**Figure 3.**
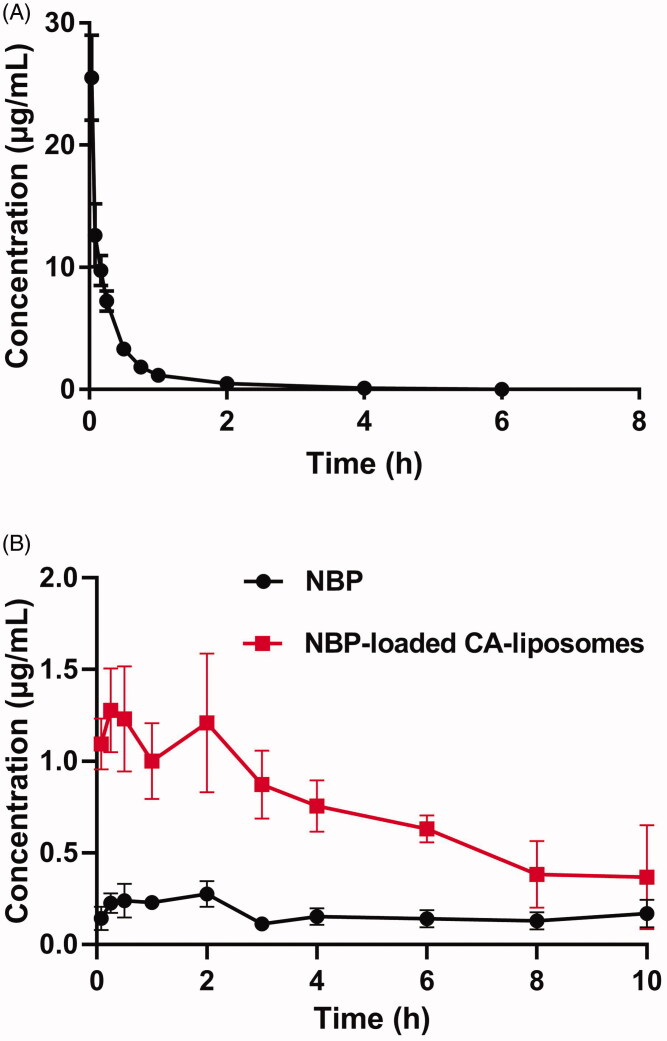
Pharmacokinetic curves of NBP after intravenous injection of NBP solution (A) and oral administration of NBP suspension as well as NBP-loaded BC-liposomes to rats (B) at a dose of 30 mg/kg, respectively. Data are mean ± SD (*n* = 5).

To investigate the pharmacokinetics of NBP and NBP-loaded CA-liposomes, they were dispersed in normal saline and orally administered to SD rats at equal doses (30 mg/kg), followed by detection of plasma NBP concentration at various times. To determine the absolute bioavailability of these formulations, the pharmacokinetic profiles of NBP after intravenous injection was monitored and the results were shown in Figure 3(A). Figure 3(B) showed that after orally received free NBP suspension, the drug absorption was very slow and weak. In addition, the maximum concentration (C_max_) (only 0.28 µg/mL) was observed at 2 h. In contrast, NBP-loaded CA-liposomes generated rapid and robust drug absorption, along with a C_max_=1.28 µg/mL within 0.25 h. Meanwhile, NBP-loaded CA-liposomes exhibited remarkably higher drug concentration compared with free NBP throughout all the period of study. Pharmacokinetic parameters calculated using plasma NBP concentration were summarized in Table 2. Specifically, NBP-loaded CA-liposomes showed considerably lower T_max_ and higher C_max_ values than free NBP (*p < .05, **p < .01). In addition, CL of drug from oral drug suspension was substantially higher than that from oral liposomes and intravenous injection of drug solution. The huge difference was possibly because oral clearance took into account the F of the formulation (Raidal and Edwards, 2008). As previously reported, the CL obtained using the software for oral administration is the apparent clearance (CL/F), where F is bioavailability after oral administration (Raidal and Edwards, 2008). Moreover, it was notable that CL of drug from oral liposomes was comparable to that from intravenous injection. This was probably due to the high absolute bioavailability of NBP-loaded CA-liposomes (92.65%), reflecting that NBP was almost completely absorbed. In addition, the relative bioavailability of NBP-loaded CA-liposomes to NBP suspension was calculated to be 427%. Therefore, NBP-loaded CA-liposomes provided excellent and quick drug absorption of NBP after oral administration, which was beneficial for efficient ischemic stroke treatment. Combined with the results of *in vitro* studies, we proposed some possible explanations for good oral absorption of NBP-loaded CA-liposomes. After oral administration, NBP-loaded CA-liposomes produce markedly higher dissolution rate of NBP in the gastrointestinal fluid than oral suspension. Subsequently, a large amount of released NBP penetrates the epithelium paracellularly and transcellularly due to the permeation-enhancing effect of phospholipids and CA (Greimel et al., 2007; Jeon et al., 2019). Therefore, it is highly suggested that enhancement of both dissolution rate and epithelium permeation ability of NBP is crucial to achieve the maximal oral drug absorption.

**Table 2.** Main pharmacokinetic parameters of NBP after intravenous injection of NBP solution and oral administration of NBP suspension or NBP-loaded CA-liposomes at a dose of 30 mg/kg, respectively (mean ± SD, *n* = 5).

Parameters	NBP (*i.v.*)	NBP (*p.o.*)	NBP-loaded CA-liposomes (*p.o.*)
t_max_ (h)	0.083	1.02 ± 0.31	0.70 ± 0.14*
C_max_ (µg/mL)	25.51 ± 3.46	0.36 ± 0.13	1.56 ± 0.44**
AUC_0-t_ (µg/mL*h)	7.59 ± 0.78	1.65 ± 0.32	7.05 ± 1.48**
CL_z_ (L/h/kg)	2.65 ± 0.25	12.11 ± 3.76	3.35 ± 1.54*
F_a_ (%)	100	21.7	92.65
F_r_ (%)	–	100	427

Notes: *p< .05, **p< .01, compared with NBP group. Abbreviations: AUC_0→t_: area under the pharmacokinetic curve throughout the study; CL_z_: Clearance rate; C_max_: maximum plasma concentration; t_max_: time taken to reach C_max_.

### 3.4. Biodistributions

**Figure 4.**
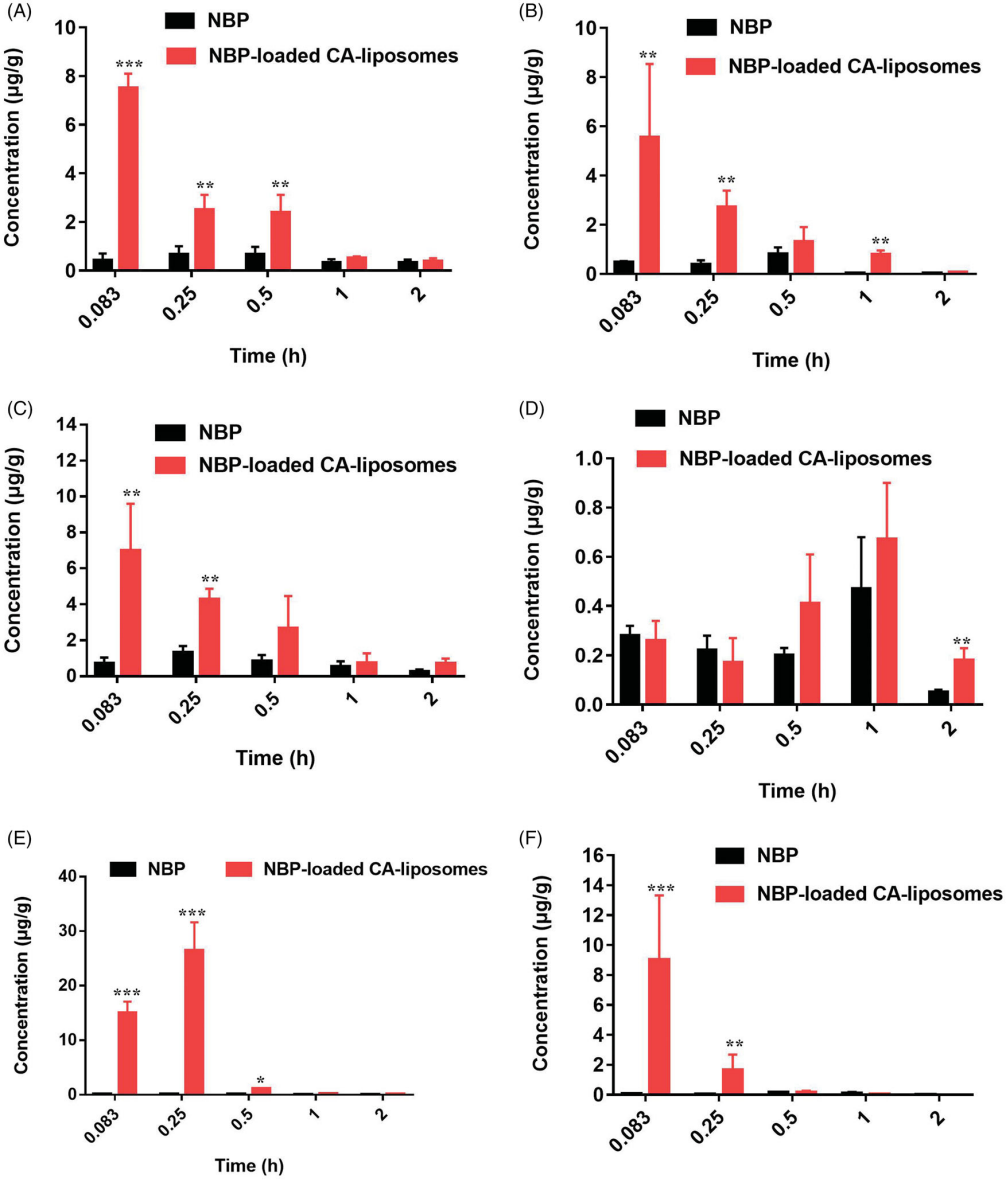
Biodistribution of NBP in major organs involving brain (A), heart (B), liver (C), lung (D), spleen (E) and kidney (F) at various time points after orally given NBP suspension and NBP-loaded CA-liposomes to rats at a dose of 30 mg/kg, respectively. Data are mean ± SD (*n* = 5). *p< .05, **p< .01, ***p< .001, compared with NBP suspension.

In addition to efficient drug absorption, achieving sufficient delivery of NBP to the brain site is another determinate factor for effective treatment through oral administration (Xiang et al., 2021). To date, oral applications of liposomes in treating brain-related disease are often failed owing to their poor BBB permeability (Agrawal et al., 2020). To investigate the accumulation capability in brain, the *in vivo* biodistribution of NBP-loaded CA-liposomes was quantitatively analyzed in rats after oral administration and shown in Figure 4. Free NBP tended to accumulate mainly in the lung while showed minimum distribution in the other organs during 2 h. In contrast, NBP-loaded CA-liposomes significantly increased drug accumulation in most organs at various time points (*p < .05, **p < .01, ***p < .001). Notably, NBP concentration in the brain of NBP-loaded CA-liposome group was 18.3-, 3.73- and 3.62-fold that of free NBP group at 5, 15, and 30 min after oral administration, indicated that NBP-loaded CA-liposome achieved a rapid and high uptake of NBP in the brain. Additionally, Re and Ce presented in Supplementary Table 5 further confirmed the profound accumulation efficiency of NBP in the brain with a rather high Re (3.48) and Ce (9.04) among the tested tissues.

### 3.5. NBP-loaded CA-liposomes demonstrated notable efficacy in MACO rats

**Figure 5.**
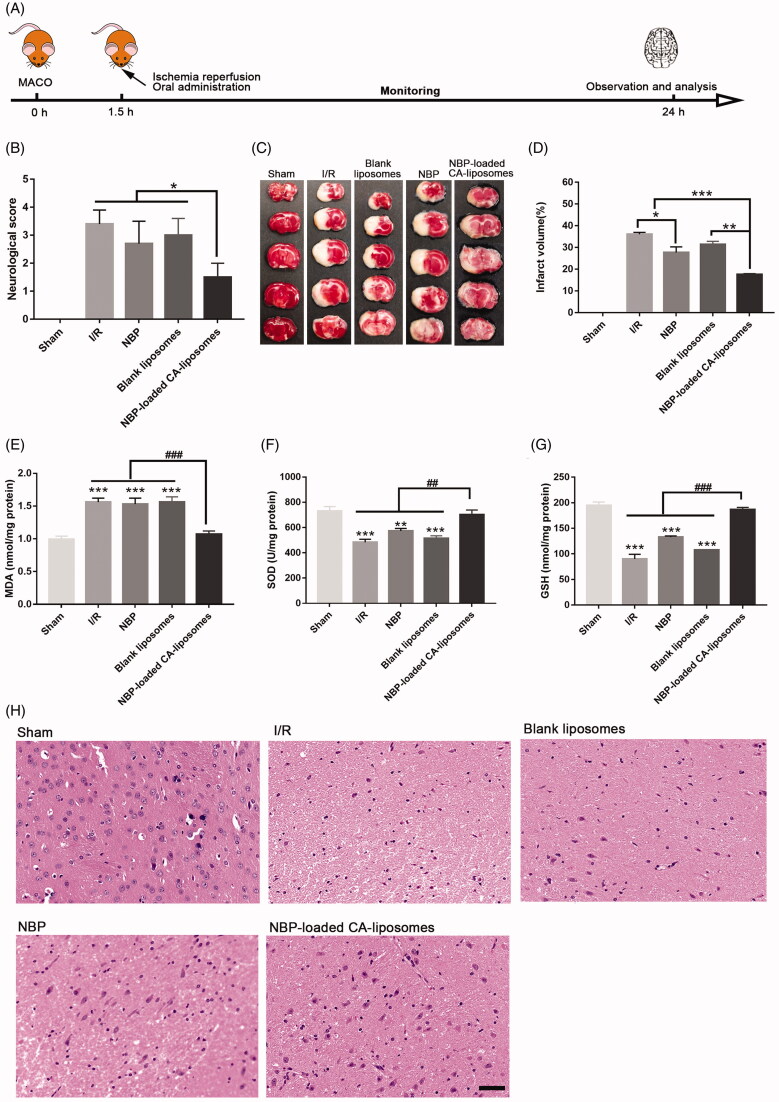
*In vivo* efficacy of NBP-loaded CA-liposomes against the cerebral I/R in rats. (A) Diagram of disease model establishment and drug-dosing regimen. (B) Neurological deficits assessed at 24 h post perfusion. (C) Representative images of infarcted brain areas indicated by TTC staining. (D) Quantitative analysis of the infarcted brain regions. The ratios of infarcts area to whole brain area were calculated. Levels of the brain MDA (E), SOD (F) and GSH (G) activity of rats 24 h after different treatment protocols. (H) H&E staining of injured cerebral hemisphere examined by a light microscope. Scale bar = 100 µm. Data in panels B and D-G are mean ± SD (*n* = 10). *p<.05, **p < .01, ***p < .001, ^#^*p* < .05, ^##^*p* < .01, ^###^*p* < .001.

The excellent brain-accumulation along with prominent oral absorption after administration of NBP-loaded CA-liposomes led us to investigate its therapeutic efficacy for ischemic stroke. As the most reliable rodent model of brain injury caused by I/R (Feng et al., 2019), the MACO model in rats was established for the pharmacodynamic evaluations. The design of animal experiments and the corresponding results were displayed in Figure 5. As shown in Figure 5(B), NBP-loaded CA-liposomes presented markedly lower neurological score (1.5) relative to I/R group (3.4) (*p < .05) while no significant difference between NBP group and I/R group was observed (*p*> .05). Consistently, the infarct volume of rats treated with NBP-loaded CA-liposomes (17.65 ± 0.34%) was significantly lower than that of I/R (36.08 ± 0.86%) and free NBP groups (27.66 ± 2.59%, ***p < .001). These results demonstrated that NBP-loaded CA-liposomes achieved superior attenuation on the MCAO-induced neurological damage than free NBP. In addition, NBP-loaded CA-liposomes significantly increased the SOD and GSH activity by 45.14% (from 482.95 to 701 U/mg) and 107.58% (from 90.01 to 186.84 nmoL/mg) as well as decreased MDA in the ischemic brains by 31.41% (from 1.56 to 1.07), compared with I/R group (*^##^p* < .01, *^###^p* < .001). To confirm the therapeutic effect, H&E staining was also performed and the results were shown in Figure 5(H). The results clearly indicated that NBP-loaded CA-liposomes produced an obvious protective effect on cerebral cells. Taken together, NBP-loaded CA-liposomes better suppressed the damage in cerebral ischemia as compared to free NBP.

## 4. Conclusions

Here, we reported on the engineering of NBP-loaded CA-liposomes as a novel oral drug delivery system for NBP that enabled much more drug to rapidly reach the brain and protect cerebral cells from damage in ischemic stroke rat model. NBP-loaded CA-liposomes demonstrated timely and profound brain-accumulation, mainly due to excellent oral absorption of NBP-loaded CA-liposomes via paracellular and transcellular diffusion across the intestinal epithelium. Our results may prompt the development of rational oral delivery platforms of NBP for efficient treatment of ischemic stroke in the clinic.

## Supplementary Material

Supplemental Material

## Abbreviations

AUC0 − t: the area under the pharmacokinetic curve throughout the study
BBB: blood-brain barrier
CA: sodium cholate
Ce: concentration efficiency
DL: drug loading capacity
EE: encapsulation efficiency
Fa: absolute bioavailability
Fr: relative bioavailability
GSH-Px: glutathione peroxidase
H&E staining: hematoxylin and esosin staining
I/R: ischemia/reperfusion
MACO: middle cerebral artery occlusion
MCA: right common carotid artery
MDA: malondialdehyde
NBP: N-butylphthalide
Re: relative uptake efficiency
SGF: simulated gastric fluid
SOD: superoxide dismutase
TEER: transepithelial electrical resistance
TEM: transmission electron microscope
