# Supplementary material for: Rapid and improved oral absorption of N-butylphthalide by sodium cholate-appended liposomes for efficient ischemic stroke therapy
Source: Drug Deliv. 2021 Nov 12;28(1):2469–79. doi: 10.1080/10717544.2021.2000678 (PMC8592624; doi:10.1080/10717544.2021.2000678)
Supplement: Supplemental Material [file IDRD_A_2000678_SM1181.docx]

**Supplementary information**

**Rapid and improved oral absorption of N-butylphthalide by sodium cholate-appended liposomes for efficient ischemic stroke therapy**

*Ailing Zhang^a,1^, Jianbo Li^b,1^, Shuaishuai Wang^c^, Yaru Xu^c^, Qinglian Li^c^, Zhe Wu^c^, Chenxu Wang^b^, Haiyang Meng ^a, *^ and Jinjie Zhang^c, *^*

^a^ Department of Pharmacy, The First Affiliated Hospital of Zhengzhou University, Zhengzhou 450052, China

^b^ Henan Key Laboratory for Pharmacology of liver diseases, Institute of Medical and Pharmaceutical Sciences, Zhengzhou University, Zhengzhou 450001, China

^c^ Henan Key Laboratory of Targeting Therapy and Diagnosis for Critical Diseases, School of Pharmaceutical Sciences, Zhengzhou University, Zhengzhou, Henan Province 450001, China

^1^These authors contributed equally to this work.

*Corresponding authors at: No. 100 Kexue Road, Zhengzhou, Henan Province, China, 450001

Email: [liger1029@126.com](mailto:liger1029@126.com) (Jinjie Zhang), [mhy_m@qq.com](mailto:mhy_m@qq.com) (Haiyang Meng)

**Supplementary tables:**

Table S1. Storage stability of NBP-loaded CA-liposomes at 4 ℃ (mean ± SD, n=3)

| Time (d) | Size (nm) | PDI | Zeta potential (mV) | EE (%) |
| --- | --- | --- | --- | --- |
| 0 | 98.11±2.37 | 0.285±0.032 | -35.1±6.14 | 93.91±0.10 |
| 2 | 106.5±3.36 | 0.289±0.007 | -34.9±0.72 | 93.81±0.27 |
| 4 | 108.8±7.10 | 0.405±0.029 | -38.1±0.76 | 93.34±1.25 |
| 7 | 92.48±3.00 | 0.384±0.059 | -37.2±2.46 | 91.22±1.17 |
| 15 | 85.52±8.64 | 0.342±0.060 | -24.7±5.60 | 89.10±0.45 |
| 30 | 81.79±1.83 | 0.478±0.030 | -15.9±5.10 | 86.47±0.43 |

Table S2. Precision and extraction recovery for NBP in rat plasma.

| Sample concentration (μg/mL) | Inter-day precision (%) | Intra-day precision  (%) | Extration recovery (%) |
| --- | --- | --- | --- |
| 2.056 | 1.38 | 1.73 | 97.27% ± 2.86 |
| 1.542 | 1.00 | 0.48 | 96.33% ± 3.41 |
| 1.028 | 0.46 | 1.41 | 97.17% ± 2.06 |

Table S3. The regression equations of NBP in serum and different tissues. (n=3)

| Sample | Linear Range (µg/mL) | Regression equation | R^2^ |
| --- | --- | --- | --- |
| Serum | 0.303-10.1 | A=14.101C+6.1226 | 0.9997 |
| Brain | 0.1-4.0 | A= 37.547C -3.57 | 0.9991 |
| Heart | 0.1-4.0 | A= 37.406C -1.1022 | 0.9992 |
| Liver | 0.08-5.0 | A = 31.469C +2.9862 | 0.9995 |
| Spleen | 0.025-5.05 | A= 14.925C + 3.7152 | 0.9995 |
| Lung | 0.1-4.0 | A= 36.322C - 0.5373 | 0.9990 |
| Kidney | 0.101-3.03 | A = 10.661C +11.3 | 0.9919 |

Table S4. Precision and Extraction recovery for NBP in rat tissues. (Mean±SD, n=3)

| Sample | Standard concentration (µg/mL) | Inter-day precision (%) | Intra-day precision  (%) | Recovery (%) |
| --- | --- | --- | --- | --- |
|  | 0.12 | 2.13 | 2.19 | 100.56±2.10 |
| Brain | 0.20 | 1.56 | 0.63 | 102.83±2.52 |
|  | 0.32 | 0.38 | 0.50 | 101.35±2.62 |
|  | 0.12 | 3.67 | 3.39 | 102.78±3.37 |
| Heart | 0.20 | 2.38 | 3.14 | 103.33±2.89 |
|  | 0.32 | 1.42 | 2.47 | 99.38±0.83 |
|  | 1.00 | 4.86 | 3.19 | 98.67±2.08 |
| Liver | 2.50 | 2.71 | 2.80 | 100.67±1.29 |
|  | 4.00 | 1.51 | 2.07 | 103.68±0.63 |
|  | 1.02 | 4.04 | 3.53 | 109.33±3.78 |
| Spleen | 2.04 | 3.75 | 2.46 | 102.67±3.25 |
|  | 4.08 | 2.39 | 2.23 | 102.50±1.89 |
|  | 0.61 | 3.15 | 2.29 | 101.67±4.41 |
| Kidney | 1.22 | 4.05 | 2.24 | 97.78±1.27 |
|  | 2.44 | 3.12 | 2.13 | 101.11±3.78 |

Table S5. Key targeting parameters calculated from the pharmacokinetic parameters of major tissues obtained in biodistribution studies. Re and Ce represent the relative uptake efficiency and the concentration efficiency, respectively.

| Organs | Re | Ce |
| --- | --- | --- |
| Serum | 4.38 | 10.56 |
| Brain | 3.48 | 9.04 |
| Heart | 4.98 | 7.28 |
| Liver | 3.08 | 5.02 |
| Spleen | 35.30 | 188.86 |
| Lung | 1.50 | 1.57 |
| Kidney | 9.24 | 47.53 |
